# The Impact of Side-Selective Laser Tailoring of Titania Nanotubes on Changes in Photoelectrocatalytic Activity

**DOI:** 10.3390/mi14020274

**Published:** 2023-01-20

**Authors:** Katarzyna Siuzdak, Jakub Wawrzyniak, Łukasz Haryński, Zuzanna Bielan, Katarzyna Grochowska

**Affiliations:** Centre for Plasma and Laser Engineering, Institute of Fluid-Flow Machinery Polish Academy of Sciences, Fiszera 14 St., 80-231 Gdansk, Poland

**Keywords:** titania nanotubes, laser treatment, electrochemical activity, structural changes, morphological changes

## Abstract

Over the last few decades, titanium(IV) oxide-based materials have gained particular attention due to their stability, corrosion resistance, photocatalytic activity under UV light, and possibilities for modification. Among various structures, TiO_2_ nanotubes (NTs) grown on Ti foil or glass substrates and obtained through a simple anodization process are widely used as photocatalysts or photoanodes. During the anodization process, the geometry of the nanotubes (length, distribution, diameter, wall thickness, etc.) is easily controlled, though the obtained samples are amorphous. Heat treatment is required to transform the amorphous material into crystalline material. However, instead of time- and cost-consuming furnace treatment, fast and precise laser annealing is applied as a promising alternative. Nonetheless, laser treatment can result in geometry changes of TiO_2_ NTs, consequently altering, their electrochemical activity. Moreover, modification of the TiO_2_ NTs surfaces with transition metals and further laser treatment can result in materials with unique photoelectrochemical properties. In this regard, we gathered the latest achievements in the field of laser-treated titania for this review paper. We mainly focused on single structural and morphological changes resulting from pulsed laser annealing and their influence on the electrochemical properties of titania. Finally, the theoretical basis for and combination of laser- and metal-modifications and their impact on the resulting possibilities for electrochemical water splitting are also discussed.

## 1. Introduction

The dynamic evolution of nanotechnology offers a variety of tools for manipulating matter at the molecular and even atomic levels [1]. Even when the chemical formula of a material is known, a change in its shape or structure can provide completely different properties and functionalities; titanium dioxide can be regarded as an ideal example. This material can take the shape of different nanoforms—particles [2,3], pillars [4], tubes [5,6,7], and a core-shell [8]—through the use of sophisticated wet-chemistry methods (hydrothermal) or physical techniques, e.g., laser ablation [9], evaporation, and atomic layer deposition [10]. Recently, approaches regarded as green synthesis [11] are also becoming increasingly popular. Due to its unique shape and size, as well as its crystalline phase, titania can find applications in biomedicine [12,13,14], photocatalysis, catalysis [15,16], solar cells, supercapacitors [17] and sensors [18,19,20], even though it is still titanium dioxide.

Given the cost and availability of the minerals ilmenite and rutile, the common use of titanium dioxide in different fields is not surprising [21]. In the form of highly ordered nanotubes (TNTs), Among other uses, TiO_2_ in the form of highly ordered nanotubes (TNTs) has gained considerable attention due to the well-controlled anodization process that is commonly recognized in technology for the formation of an anticorrosive, protective, thin oxide films over the metallic substrate [22]. The whole experimental setup is simple and is based on a two-electrode arrangement in which Ti serves as the anode and a Pt or carbon rod serves as the cathode, both immersed in an electrolyte containing fluoride ions [23].

This method offers morphological control via changes in the applied potential, electrolyte composition, temperature, and process duration [24]. The whole laboratory setup is simple and does not require advanced machines, which makes this method increasingly popular for the fabrication of nanomaterials. It should be underlined that tubular structures grow directly out of the Ti substrate and no further immobilization process is required for application, e.g., in electrochemical water splitting. Moreover, the method can be applied to titanium of different shapes, from flat plates and wire [25] to screws and semitransparent substrates (e.g., indium tin oxide) coated with a layer of Ti [26]. The ability to form an ordered nanostructure over large areas [27] is also beneficial for further commercialization. Such an organized geometry provides a highly developed surface area that is available for modification, e.g., the deposition of a conducting polymer [28], or for decoration by metal or metal oxide nanoparticles [29]. Moreover, TNTs can be used as a reservoir for drug molecules, which can be released due to the concentration gradient or an external stimulus [30]. One can find many examples of the utilization of titania nanotubes in the literature, not as a particular chemical compound but regarded as an ordered and stable scaffold for other materials [31].

However, many of the applied methods for modifying TNTs require the usage of chemicals that are problematic to dispose of. The process upscaling requires several optimization steps, and the elaborated route can only be performed at the laboratory scale and cannot be carried out on only a specific part of the substrate. In contrast to these limitations, laser technology offers rapid and easily scalable processing based on the interaction between the coherent, intense light of a particular wavelength with the material [32,33,34,35,36,37,38,39]. Additionally, the selection of the laser fluence and the environment in which the process is carried out (vacuum, ambient atmosphere, or ever water) can also be included into the group of important parameters affecting the final product [39,40] (see Table 1).

As can be seen, the modification of TiO_2_ NTs with laser radiation can be implemented using several different lasers which emit wavelengths from almost the whole spectrum. In most cases, the process leads to an amorphous-to-crystalline phase transition with a multi-fold increase in the measured photocurrent as well as a general enhancement of catalytic activity [35,40,41,42,43]. However, several drawbacks of the used treatment were also described. In the work of Xu and Zangari [35], after increasing the laser fluence above 0.3 J/cm^2^, the amount of photoinduced charge carriers dropped significantly, resulting in a final decrease in the activity of the samples. Moreover, both Xu et al. [40] and Wawrzyniak et al. [43] observed an increment of formed structural defects, leading to a decrease in activity.

Herein, we present the selective modification of titania nanotubes using radiation generated by a pulsed Nd:YAG laser (Quantel, Lannion, France). The aim of this work is focused on the impact of the laser beam on the tubular surface to achieve phase conversion, change the surface morphology, and integrate the TNTs with other metal or metal oxide species. In the presented experiments, the selected laser offered four different harmonics and a wide range of fluences that provided the variety of options selected for the experiments. Nonetheless, particular attention was paid to modification using the laser operating at 355 nm. This is due to the fact that this is the only wavelength of an Nd:YAG laser that produces energy (3.5 eV) close to the value of the titania bandgap (3.2 eV), and therefore contributes the highest absorption probability.

**Figure 1:**
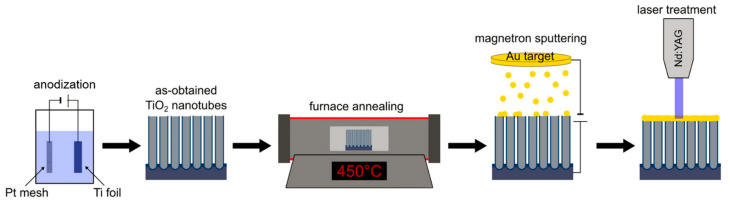
Illustration of protocol to obtain laser-modified TiO_2_ NTs, based on Au-decorated TiO_2_ NTs. Reprinted with permission from [45]. Copyright (2022) Creative Commons Attribution.

The TiO_2_ NTs described were obtained via two-electrode electrochemical oxidation, in which a platinum mesh functioned as the cathode and Ti foil functioned as the anode. Various process parameters such as applied voltage, anodization time, temperature, and electrolyte composition were used to obtain nanotubes with different geometric features (e.g., length and separation). After the anodization process, the samples were washed in ethanol to remove the remaining electrolyte, dried, and calcined at 450 °C for 2 h within the 2 °C/min heating range. Modification with transition metals was performed using magnetron sputtering. Finally, laser treatment of the obtained samples was performed using energy fluence values in the range of 10–80 mJ/cm^2^. A general illustration of the sample-obtaining steps is presented in Figure 1.

During laser treatment, the substrate was placed on a motorized stage to enable the precise change of position between the laser beam and the moving substrate, which made it possible to modify a selected point of the material. In our studies, the layer of titania nanotubes was annealed directly after the anodization or calcination and magnetron sputtering step, ensuring crystallization, the melting of the topmost region, and the selective encapsulation of the nanotubes. Regarding the latter cases—before laser annealing, a thin metallic film was also sputtered onto the titania surface and treatment with the laser radiation was performed afterward. These approaches lead to significant changes not only in morphology, as was verified by scanning electron microscopy, but also totally different activities of the final material, especially toward photoconversion and water-splitting processes. A detailed inspection and various optimization approaches showed the promising application of laser radiation toward the precise treatment of ordered titania nanotubes and that manipulation at the nanoscale still offers various possibilities.

Following this line of thought, we centered our selected research efforts for this review on the prominent impact of nanosecond laser treatment on TiO_2_ nanotubes’ structural and morphological changes, resulting in an increment in their electrochemical properties. As a part of an increasing trend, recent, worldwide novelties in this topic were also mentioned and discussed. We would also like to underline that we found this method interesting because no chemical compounds or waste disposal, which can make this process problematic for large-scale production, are required. In particular, it is worth mentioning that we were able to successfully close separated nanotubes, obtaining a hollow interior, which has not been achieved until now using other technologies. Such materials offer a promising strategy for broadening the application of TiO_2_ nanotubes in various fields. We hope to provide interested readers with a platform for future research.

## 2. Theoretical Studies on the Effect of Laser Irradiation on Titania Nanotubes

To investigate the influence of a laser beam on TNTs, theoretical studies were conducted. For the very first time, the work of Kupracz et al. [46] demonstrated simulations of temperature dispersion inside nanotubes during laser heating through implementing the finite element method (FEM). It is well-known that the heat transfer during laser treatment can be described by the heat flow equation [47]:

$$\rho C\left(T\right)\frac{\delta T}{\delta t}=\kappa \left(z,\;T\right)\frac{\delta^{2}T}{{\delta z}^{2}}+\alpha I\left(z,t,\;T\right)$$

where T, C, ρ, κ(z,T) and α are the temperature, heat capacity, density, thermal conductivity, and light absorption coefficient, respectively. I(z,t,T) is the laser beam intensity at depth z and the time that has passed since the beginning of the laser pulse. Nonetheless, several assumptions must be made to perform such calculations. Concerning the geometry of the nanotubes, the fact that the wall thickness increases with the nanotube length—meaning that the shape of the nanotubes is not ideally cylindrical, but that the cross-section area increases from the top to the bottom of the nanotubes—should also be taken into account [48]:

$$\mathrm{rr}\;\left(z\right)={\mathrm{rr}}_{0}+\left(\frac{D}{2}-{\mathrm{rr}}_{0}\right)\cdot \exp\left(\frac{-H-z}{D/2}\right)$$

where rr and rr_0_ are the nanotube wall thickness and minimum wall thickness, respectively, while D and H stand for the diameter and height of the nanotubes, respectively [46]. This is of key importance, as the heat generation and its dissipation are correlated with the TNT thickness. A more detailed description of the proposed model can be found in [46], and a thorough discussion on the laser’s interaction with titania can be found in [49].

Furthermore, during the simulations, the change in the wall thickness (5–20 nm) was not the only detail to consider. The length of the nanotubes (0.5–2 µm) was also considered. No other imperfections of material geometry were considered (e.g., cracks or ribs) that could also induce local variations of the thermal properties. It should be mentioned that the simulations were performed for polycrystalline anatase nanotubes placed in a vacuum with fixed-laser working parameters including a fluence of 25 mJ/cm^2^, a pulse duration of 6 ns, and a wavelength of 355 nm. As defects are created during the heating of the titania material—namely, oxygen vacancies and a reduction of Ti^4+^ to Ti^3+^ [50]—the reduction of the optical bandgap with the temperature was also included.

**Figure 2:**
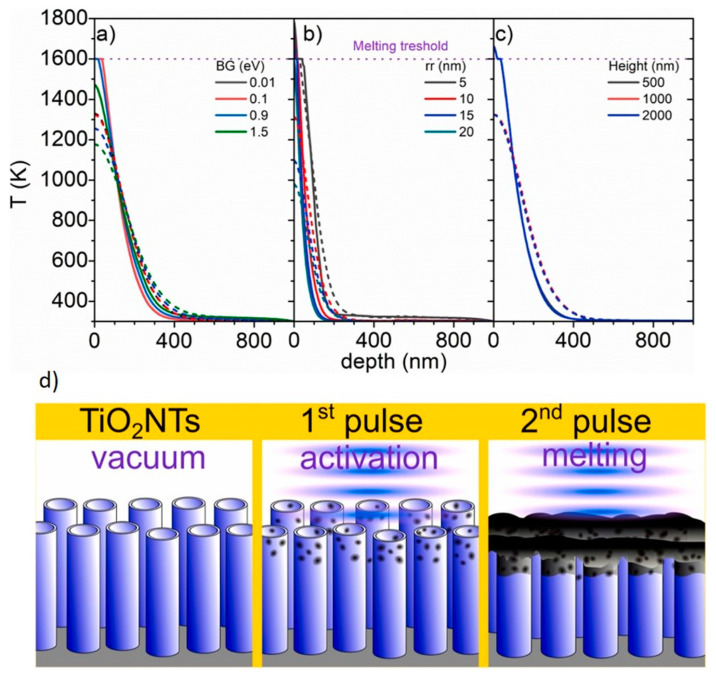
Temperature distribution in laser-treated TiO_2_ nanotubes as a function of (**a**) optical bandgap (BG), (**b**) initial wall thickness, and (**c**) height. (**d**) Evolution of titania material morphology during laser annealing. Reprinted with permission from [46].

It was first established that, after the first pulse, the heat generation in the material is barely enough to induce a temperature increase up to 560 K. The second and subsequent pulses lead to a temperature increase up to ca. 1400 K at the top of the TNTs; this decreases with the length of the nanotubes to 560 K at a depth of 190 nm. It should be kept in mind that the melting threshold for titania nanotubes is 1600 K [51]. However, changing properties of the material, such as the value of the optical bandgap or the initial wall thickness, can result in the top of the TNTs heating up to temperatures higher than those designated by the above-mentioned criterion, while the maximum simulated temperature is independent of the nanotube’s length (Figure 2). Moreover, it was proven that, during laser treatment, the temperature needed for the anatase-to-rutile phase transition can be achieved. Nonetheless, it should be underlined that the heat generation ratio, thermal conductivity, and repetition rate (time between consecutive laser pulses) should also be taken into account to fully investigate the evolution of the TNTs’ morphology and phase transition. Overall, the obtained results of the FEM simulations are in agreement with experimental works, which are shown later in the text.

## 3. The Laser-Induced Phase Transition of Titania Nanotubes and Its Impact on the Photoelectrocatalytic Activity

As mentioned above, titania nanotubes may undergo a phase transition after laser annealing. Indeed, in 2008, a local, amorphous anatase TNT transformation after exposure to laser irradiation originating from a Raman spectrometer equipped with an Ar^+^ ion green laser (514.5 nm, power of 6 mW·µm^−2^) was reported by Likodimos et al. [52]. The TiO_2_ nanotubes were locally heated up to 700 K during laser irradiation and the whole exposure lasted 1 s.

**Figure 3:**
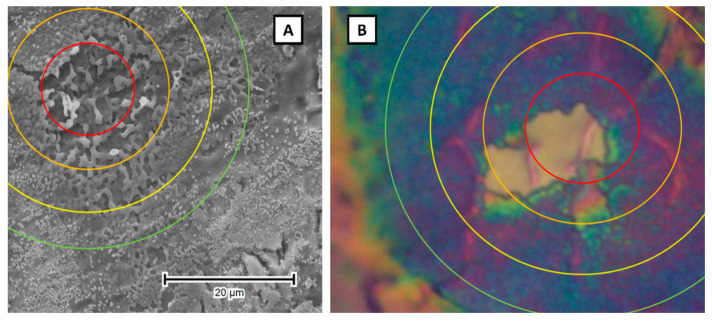
SEM (**A**) and optical (**B**) image of the hotspot of laser-modified titania nanotubes with the size of influence indicated.

The effectivity of the laser-induced TNT phase transformation depends on many factors, such as the pulse duration, frequency, laser type, fluence, atmosphere inside the vacuum chamber, and morphology of the starting material [49]. Initially, during the preliminary investigation phase, we treated the titania nanotubes with a homogenized laser beam in stationary mode, without the relative movement of the laser beam and the sample. As a result, we observed an array of hotspots on the modified surface, each surrounded by a slightly different zone of influence centered in a particular hotspot. Unfortunately, it meant that, laterally, the samples varied greatly in crystallinity and degree of modification (Figure 3). In the following stages, a motorized table was employed to move the sample during consecutive laser pulses in the selected direction, which made it possible to unify the degree of modification over the whole area of the sample. Moreover, due to the programmability of the motor’s movement, the modification of specific shapes became an option.

**Figure 4:**
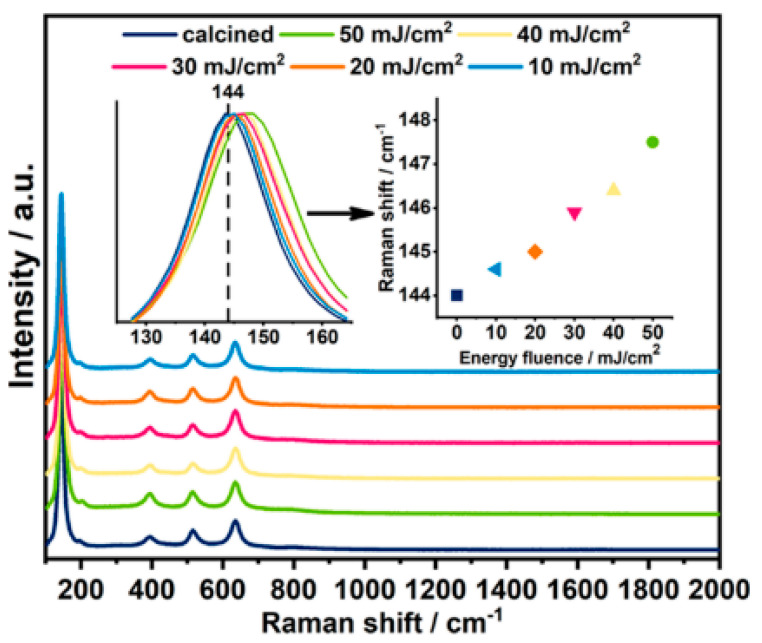
Raman spectra recorded for laser-treated TNTs in comparison to a calcined sample. Inset (a): dependence of the E_g(1)_ position on the utilized laser energy fluences. Reprinted with permission from [53].

In the work of Haryński et al. [53], the impact of a 355 nm Nd:YAG pulsed laser fluence (in the range of 10–50 mJ/cm^2^, in a vacuum atmosphere) on the crystalline phase of previously calcined titania nanotubes was studied, resulting in the enhancement of their photoactivity. While the 10 mJ/cm^2^ fluence did not cause significant changes in the material structure, the higher energy fluences caused melting in the tops of the TNTs; the thickness of the melted layers was ca. 200 nm. This is consistent with the previously presented theoretical predictions [46]. Moreover, the blue shift of the main E_g(1)_ active anatase peak was observed (Figure 4) and was ascribed to the presence of oxygen vacancies. It can be observed that the higher the fluence, the more shifted the E_g(1)_ mode was, while other peaks corresponding to the anatase phase were located at the same position for all laser fluences. Additionally, no signal from rutile was observed.

**Figure 5:**
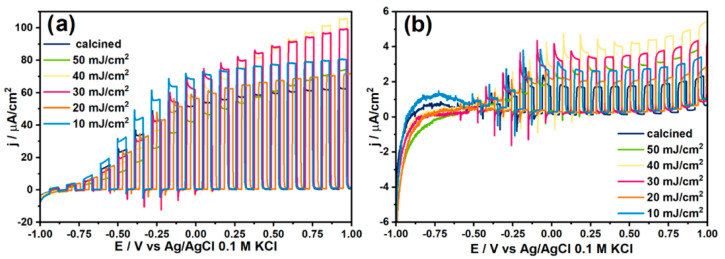
Linear voltamperograms of laser-modified anatase TNTs in comparison to a calcined sample carried out in 0.5 M of Na_2_SO_4_ electrolyte in UV-vis (**a**) and vis (**b**) light. Reprinted with permission from [53].

Nonetheless, the conducted research also indicated the presence of additional laser-induced shallow states originating from the degradation of the crystalline structure. Those shallow states, along with the oxygen vacancies, were responsible for the increased photoelectrochemical activity of the laser-modified anatase TNTs (Figure 5). The photocurrent densities registered for all the samples increased with the applied potential. Moreover, it can be seen that, in the fluence range of 10–40 mJ/cm^2^, the response of the modified TNTs increased in comparison to the calcined TNT, with a maximum increment of ca. 45% for the 40 mJ/cm^2^ fluence. For an n-type semiconductor, the depletion layer grew with the applied potential; nonetheless, for tubular structures, the saturation of the photoresponse was expected. In our case, the growth of the response observed was due to the presence of a melted layer at the top of the TNTs, meaning that the material could be treated as a compact layer [54].

Taking into account the results for UV-vis and only visible light, one can establish the optimal laser-processing parameters to achieve the highest photoresponse.

**Figure 6:**
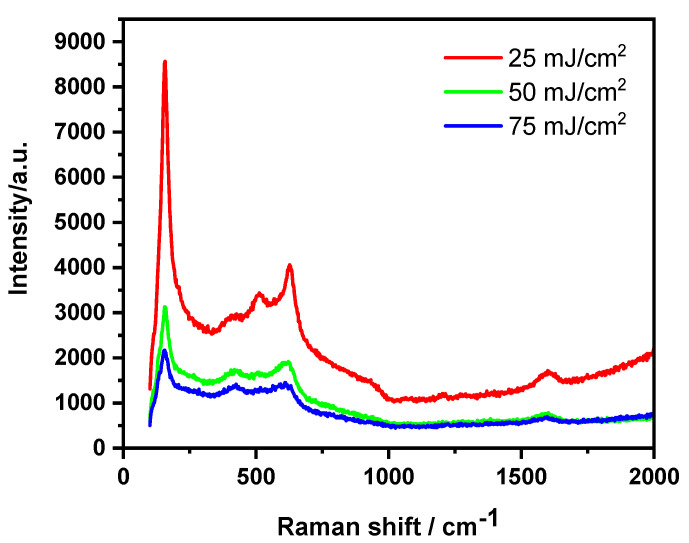
Raman spectra recorded for laser-treated amorphous TNTs. Reprinted with permission from [55].

In our next work [50], the influence of laser treatment on amorphous titania material was studied. An Nd:YAG laser operated at a wavelength of 266 nm with a fluence in the range of 25–75 mJ/cm^2^ was used, and the process was carried out in an ambient-air atmosphere. We were able to detect anatase peaks for all the used fluences; nevertheless, the intensity of the peaks decreased with the increasing laser beam energy (Figure 6), which was attributed to the laser-induced disordering of the structure due to rapid temperature changes. Moreover, the E_g(1)_ mode shift was also observed, as for laser-treated anatase TNTs.

**Figure 7:**
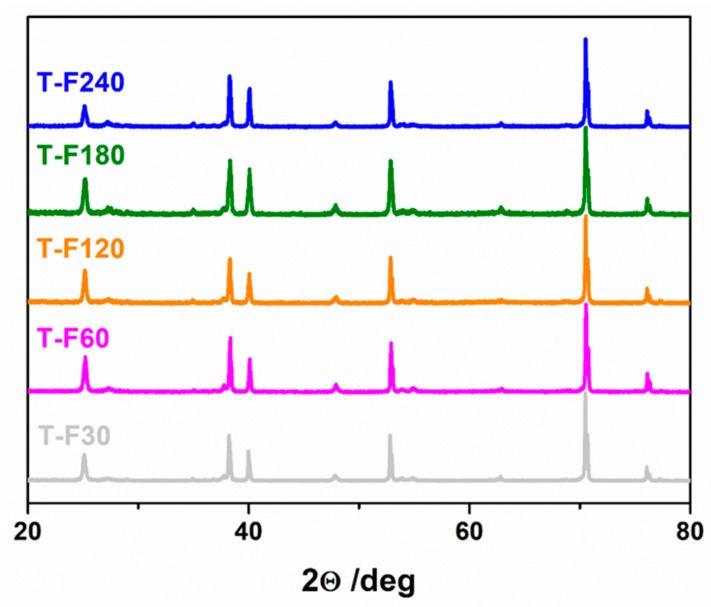
XRD patterns for calcined TiO_2_ nanotubes treated with different laser fluences. Reprinted with permission from [45]. Copyright (2022) Creative Commons Attribution.

To thoroughly investigate the influence of laser irradiation on calcined titania nanotubes, X-ray diffraction measurements (XRD) of pristine TiO_2_ NTs treated with different laser fluences are presented in Figure 7. The detected patterns can be mainly ascribed to anatase (25.26°, 48.01° and 55.13°, PDF-2 01-071-1168) and titanium (35.09°, 38.43°, 40.19°, 52.98°, 63.01°, 70.67° and 76.29°, PDF-2 00-044-1294). Small peaks from rutile are also visible (27.44° and 54.16°, PDF-2 01-076-0320). The UV-laser processing led to the decrement of the anatase-to-titanium ratio, and the Ti (103) peak (70.67°) to the Ti (002) peak (38.43°) ratio. This corresponds to the shortening of length of the nanotubes, as the Ti plane (103) is dominant for the structure of the nanotubes. Moreover, the laser treatment can lead to some degradation of the crystal structure.

**Figure 8:**
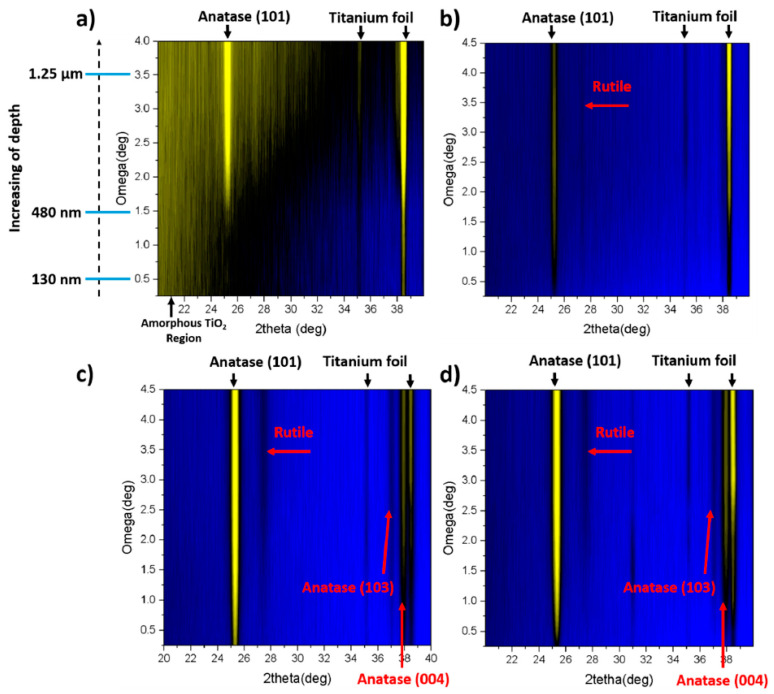
Gi-XRD-2D image scans of amorphous (**a**,**b**) and calcined (**c**,**d**) TiO_2_ nanotubes annealed by laser in air (**a**,**c**) and vacuum (**b**,**d**). Reprinted with permission from [56].

Going further, the differences in the structure of as-anodized and calcined TNTs undergoing laser annealing are best represented by the grazing incident XRD (Gi-XRD) technique. This technique graphically represents changes in crystallinity along the nanotube from the top to the bottom. As can be seen in Figure 8, for an amorphous material treated in air, the top of the TNTs remained amorphous up to ca. 480 nm down the tube, due to the rapid melting and resolidification. From that depth, the anatase phase became more visible. In the case of a vacuum, the anatase phase was observed along the entire length of the TNTs. Moreover, a weak signal assigned to rutile was detected, which can be explained by the fact that crystallization in a vacuum is more efficient. For the calcined samples, both in the air and in a vacuum, the anatase peak was pronounced, and a weak signal from rutile was also seen. Additionally, for all the prepared materials, the intensity of the anatase peak increased with the penetration depth.

Overall, the presented results confirmed that a phase transition during laser processing is possible. Moreover, laser annealing makes it possible to perform modifications over a strictly selected area and under optimized conditions. This technique can be regarded as superior to traditional, energy- and time-consuming furnace treatment [39].

## 4. The Laser-Induced Morphological Transformation of Titania Nanotubes and Its Impact on Photoelectrocatalytic Activity

Beyond the phase transition, laser radiation can also have a profound effect on the geometry of titania nanotubes. This depends not only on the type of laser, wavelength, and energy used but also on the original morphology and crystalline structure of the material being modified.

**Figure 9:**
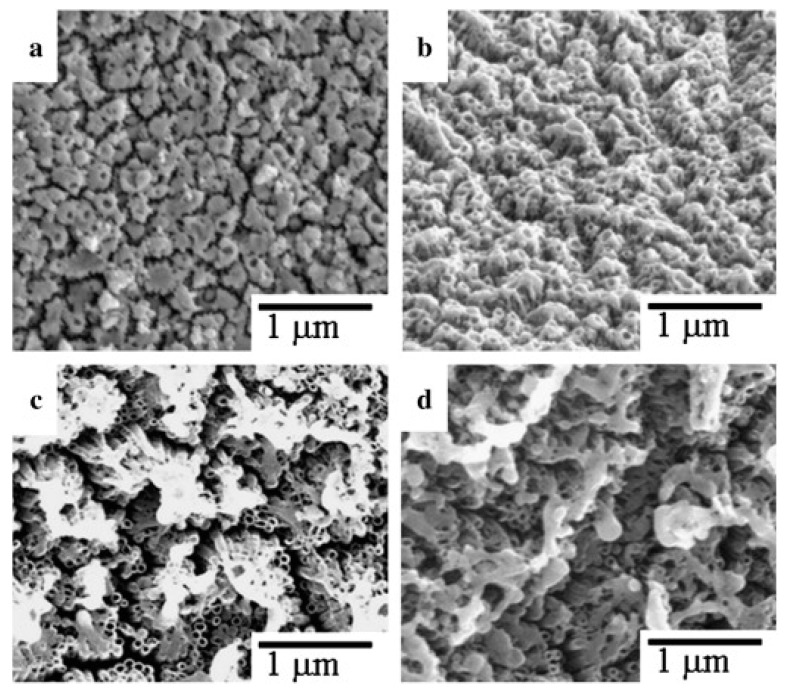
Surface of titania nanotubes irradiated with 9000 bursts at 125 mJ/cm^2^ at a 0° (**a**), 30° (**b**), 75° (**c**), and 85° angle (**d**). Reprinted with permission from [57].

One of the first works dedicated to investigating the effects of laser treatment on ordered titania arrays was published by Hsu et al. [57], who used excimer pulsed radiation (246 nm, 67–400 mJ/cm^2^, pulse duration: 25 ns) to change the geometric features of amorphous titania on a tilted plate. With this approach, Hsu et al. observed a melting of titania nanotubes in the upper region, and their reformation in groups. The effect of the modification in a tilted mode was similar to irradiation on a flat surface, except with a lower fluence; the greater the tilt, the lower the degree of modification (Figure 9).

**Figure 10:**
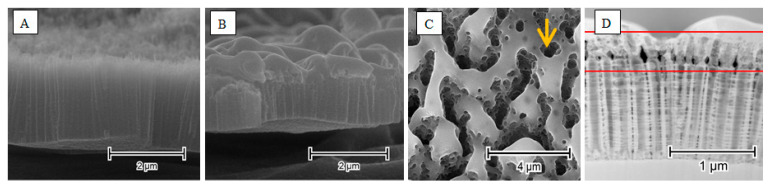
SEM images of as-anodized titania nanotubes (**A**), and nanotubes after laser-treatment (**B**). TEM images of hollow nanopillars from the top (**C**) and side (**D**). The arrow indicates the direction of laser beam. Reprinted with permission from [55].

Using an Nd:YAG pulsed laser (wavelength: 266 nm, 25–75 mJ/cm^2^, pulse duration: 6 ns) on crystalline nanotubes with a similar morphology, we found that athough the remelting of the upper nanotube region occurred, it formed a rather uniform structure covering the whole irradiated area. Only very specific laser parameters (30° tilt, 50 mJ/cm^2^ fluence) produced a microporous structure in which the pores would align toward the laser beam, whereas those modified with 75 mJ/cm^2^ formed a very characteristic neck underneath the resolidified layer (see Figure 10D within the region limited by the horizontal lines). In this process, up to 60% of the nanotube height was re-integrated into the upper layer, whereas a greater tilt resulted in a lower loss of nanotube length (Figure 10) [55].

**Figure 11:**
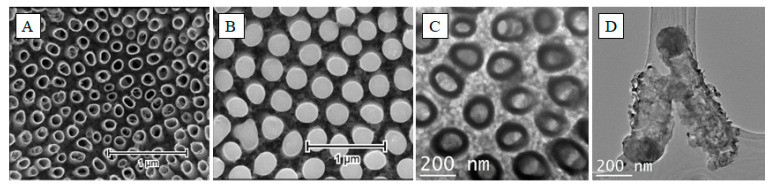
SEM images of as-anodized titania nanotubes (**A**), and nanotubes after the laser-treatment (**B**). TEM images of the hollow nanopillars from the top (**C**) and side (**D**). Reprinted with permission from [43].

However, the effects of irradiation vary greatly when it is applied toward a free-standing (or self-standing) nanotube morphology [43]. Due to of the spacing between the nanotubes, the melted upper part cannot connect to the neighboring nanotubes; therefore, a uniform layer cannot form on top of the structure. Instead, the melted material collapses inside the nanotube opening, forming a tight seal. Factors that are critical to the formation of such structures are the wavelength of the used laser, the energy of the pulse, and the distance between the nanotubes. By using 355 nm radiation at a fluence of 30 mJ/cm^2^ on nanotubes separated by about 100 nm, we can create individual, hollow titania nanopillars (Figure 11).

It was proven that appropriately adjusted laser parameters can enhance the photoactivity of titania nanotubes [40,53]. However, laser treatment can also be used successfully to enhance the catalytic activity toward an oxygen evolution reaction (OER) in titania-based electrodes. As transition metals and their oxides are a promising, inexpensive alternative to the platinum-group metal species when it comes to catalysis, much research is being performed to showcase their feasibility in this area [58,59,60,61].

**Figure 12:**
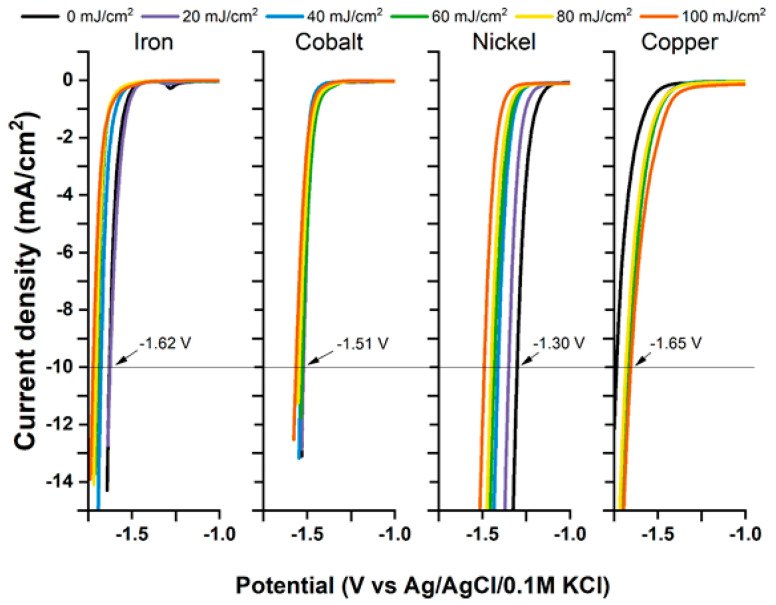
Potentials of hydrogen evolution reaction for metal-modified and laser-treated samples. The HER potential for unmodified nanotubes is −1.72 V. Reprinted with permission from [63].

Recent studies also underlined the effects of transition-metal deposition on free-standing titania nanotubes before and after laser treatment in hydrogen and oxygen evolution reactions [62]. In brief, the investigated samples were prepared by the anodization of a titanium foil in a diethylene glycol-based electrolyte. Calcination at 450 °C occurred next, and this was followed by the deposition of a 5 nm magnetron-sputtered metal layer and pulsed laser treatment (λ = 355 nm). For all four of the investigated metals (Fe, Co, Ni, and Cu), simply sputtering a thin metal layer on top of the nanotubes was enough to lower the overpotential required for hydrogen evolution reaction—0.1 V for iron- and copper-, 0.2 V for cobalt-, and 0.4 V for nickel-sputtered samples (Figure 12).

**Figure 13:**
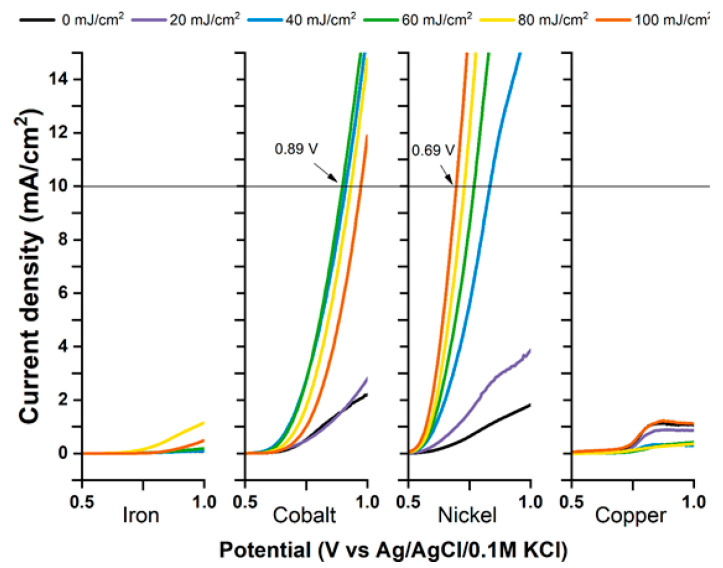
Potentials of oxygen evolution reaction for metal-modified and laser-treated samples. Unmodified TNTs are inactive toward OER within the measured range. Reprinted with permission from [63].

Interestingly, applying the laser treatment elevates the overpotential values of HER. Exactly the opposite is true for the oxygen evolution reaction. The metal deposition itself does not increase the current density unless the samples are also laser-treated. Although only a relatively small fluence is required (20–30 mJ/cm^2^) to multiply the generated current density, the cobalt-covered electrode performed best when irradiated with 60 mJ/cm^2^, showing an overpotential of 0.75 V, whereas the performance of the electrode containing nickel rose gradually up to 100 mJ/cm^2^, at which point its OER overpotential was at 0.55 V (Figure 13).

Further investigation of the Mott–Schottky plot revealed that with an increased fluence of the laser treatment, the number of charge carriers in the electrode increases, and that it increases by an additional two orders of magnitude for some time after it has been submerged in an electrolyte. An analysis of X-ray photoelectron spectra of the samples, taken after each modification step—namely, sputtering, laser-treatment, and electrochemical measurements—explains the behavior of the electrodes. In the process, the initially metallic layer transforms into oxides after submersion in the electrolyte—oxyhydroxides—which can form both from cobalt and nickel, but not from iron and copper. It should also be mentioned that laser treatment also results in changes in the morphology. Additionally, above some fluence values, the open ends of the nanotubes become tightly capped; this happened with TNTs treated without a metallic film.

**Figure 14:**
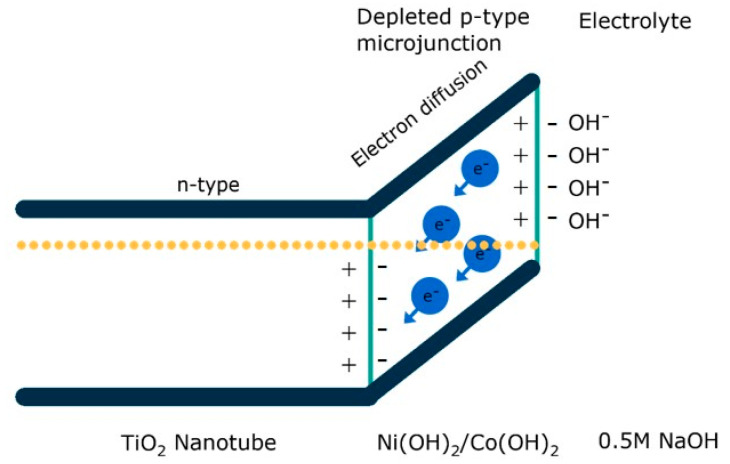
Schematic representation of the p-n microjunction facilitating the oxygen evolution reaction in electrodes containing nickel and cobalt oxides.

Taking into account the gathered results—especially the X-ray photoelectron spectroscopy and electrochemical measurements—a mechanism of charge transfer in the prepared materials was proposed. The affinity for oxyhydroxides facilitates the adsorption of OH^−^, which then aids in oxygen generation. Furthermore, nickel [64] and cobalt [65] oxides are considered p-type semiconductors, whereas TiO_2_ is an intrinsic n-type semiconductor [66], as are iron [67] and copper [68] oxides. This indicates that a p-n junction can form at the interface between the sputtered metal and the titania nanotubes underneath when cobalt and nickel oxides are present as metal species in the upper region of the TNTs. Consequently, as the electrons leave the thin, metal oxyhydroxide layer, it depletes, gaining a more positive charge relative to the titania, which in turn allows for a much easier adsorption of the oxygen from the OH^−^ groups (Figure 14). Moreover, laser modification can result in the formation of additional oxygen vacancies within titania, leading to an elevated potential difference in the depleted micro p-n junction [69,70].

Summarizing this part of the work, we have proven that laser modification can lead to changes in nanotube geometry, especially in terms of sealing laterally spaced tubes. Combined with laser annealing, the additional decoration of such material results in improved activity toward OER, while the opposite trend is observed toward HER.

Due to their unique and highly appreciated photoelectrochemical activity, laser-treated titania nanotubes further modified with a thin metallic layer, could be used not only in water-splitting reactions but also as the active material in various processes, including photocatalysis, the degradation of pollutants in the liquid or gas phase, and antibacterial treatment when combined with silver or copper [71]. Nevertheless, several limitations of the described laser-processed material also need to be considered. The laser treatment itself is not sufficient to modify TiO_2_ NTs to enhance their activity in the visible light range. In this regard, an additional modification, such as with metal nanoparticles, the introduction of intrinsic defects, or the formation of a heterojunction is needed for visible light range activity. Moreover, the selection of the laser treatment parameters is one of the most significant challenges. The selection of improper process parameters may cause sample destruction or the intended material properties will not be achieved.

## 5. Conclusions

Herein, we presented the most recent and unique approaches to the laser modification of titania nanotubes, including both theoretical and experimental research. In our review, we demonstrated the theoretical predictions for laser interactions with nanotubes, taking into account the geometric features of the material, such as wall thickness and length. Such simulations, with the use of the FEM method, were reported for the first time. It was also proven that, under optimized conditions, both phase transformation and the melting of the TNT tops can be achieved. This is in agreement with the experimental data collected subsequently. Laser processing induced a phase conversion from amorphous to crystalline using an ND:YAG laser operating at a wavelength of 355 nm. For in-depth studies, the Gi-XRD technique was applied, which made it possible to assess the crystallinity of the nanotubes along their length. Such investigations were also performed for the first time for TNTs treated with laser radiation. Laser annealing could also be exploited toward closing nanotubes while ensuring that the tube interiors remained hollow. Until now, this type of highly selective treatment, which can bring completely novel applications, has not been achieved by another method. In a further step, as a consequence of depositing a thin metallic layer combined with laser processing, the obtained material exhibited improved photocatalytic activity toward OER; the activity was incomparably higher when compared to the substrate’s activity before treatment. Moreover, using a homogenized laser beam and a motorized table facilitated the scaling of the proposed route from the laboratory to the commercial production level. As laser processing makes it possible to treat a well-defined area of a desired shape, it opens new doors in the field of materials engineering and is supported by investigations at the level of basic science.

For greater readability, the main research achievements described in the review article are also summarized in the following points:

- A unique approach to TiO_2_ NTs laser processing was developed;
- The first FEM simulations regarding laser-treated TiO_2_ NTs were performed;
- Laser treatment as a highly selective method was successfully combined with the surface modifications of TiO_2_ NTs with transition metals;
- An in-depth discussion on the influence of the laser on the electrochemical properties of the materials was provided;
- A new way to selectively and accurately close nanotubes with a laser beam was developed.

## Figures and Tables

**Table 1:** Comparison of the selected literature on laser-treated titania nanotubes.

Used Laser	Wavelength [nm]	Achievement	Drawback	Reference
Ti-sapphire femtosecond laser	800	15-fold photocurrent enhancement and double-MB photodegradation in visible light	n.d.	[41]
KrF pulsed excimer laser	248	1.5-fold photocurrent improvement under AM1.5G solar irradiation	Decrease of photoinduced charge carriers when energy fluence was >0.3 J/cm^2^	[35]
KrF pulsed excimer laser	248	Enhanced catalytic activity	Decrease of crystallinity	[40]
Picosecond diode pumped thin-disk laser	257.5	Amorphous to anatase phase transition	More structural defects, lower photocurrent densities, lower flat band potential	[39]
Nd:YAG laser	266 and 355	Multicyclic stability and time-effective phase transition	n.d.	[42]
Nd:YAG laser	266, 355 and 532	Creation of spaced, hollow nanopillars with photonic properties	Increase of oxygen vacancies for 4ω harmonics samples	[43]
DPSS laser	532	Amorphous-to-anatase phase transition	Possibility of rutile formation	[44]

n.d.—no data; MB—Methylene Blue; DPSS—diode-pumped solid-state (laser).

## Data Availability

The raw/processed data required to reproduce these findings cannot be shared at this time due to technical or time limitations.
